# Long non-coding RNA TP73-AS1 is a potential immune related prognostic biomarker for glioma

**DOI:** 10.18632/aging.202490

**Published:** 2021-02-11

**Authors:** Bo Zhang, Qinglin Li, Bin Wu, Shuyuan Zhang, Liwen Li, Kai Jin, Sheng Li, Kai Li, Zeng Wang, Yi Lu, Liang Xia, Caixing Sun

**Affiliations:** 1 Institute of Cancer and Basic Medicine, Chinese Academy of Sciences, Hangzhou 310022, People’s Republic of China; 2 Department of Neurosurgery, Cancer Hospital of the University of Chinese Academy of Sciences, Hangzhou 310022, People’s Republic of China; 3 Department of Neurosurgery, Zhejiang Cancer Hospital, Hangzhou 310022, People’s Republic of China; 4 Key Laboratory of Head and Neck Cancer Translational Research of Zhejiang Province, Hangzhou 310022, People’s Republic of China; 5 Department of Integrative Chinese and Western Medicine, Zhejiang Cancer Hospital, Hangzhou 310022, People’s Republic of China; 6 Scientific Research Department, Zhejiang Cancer Hospital, Hangzhou 310022, People’s Republic of China; 7 Department of Medical Imaging, Zhejiang Cancer Hospital, Hangzhou 310022, People’s Republic of China

**Keywords:** glioma, TP73-AS1, immune, prognostic biomarker

## Abstract

Glioma is one of the most common primary brain tumors, and is divided into low-grade and high-grade gliomas. Long non-coding RNAs have been increasingly implicated in the pathogenesis and prognosis of glioma. Here, we demonstrated that the long non-coding RNA TP73-AS1 is differentially expressed among gliomas with different clinicopathological features in The Cancer Genome Atlas (TCGA), Chinese Glioma Genome Atlas (CGGA), and GEO glioma datasets; high expression of TP73-AS1 was associated with poor clinical features, including age, stage, IDH mutation status, 1p/19q co-deletion status and overall survival. Measuring TP73-AS1 expression using real-time PCR showed the same result for 76 glioma tissue samples from our hospital. The infiltration levels of various immune cells in the tumor microenvironment were found to be significantly higher in patients with high expression of TP73-AS1. Taken together, our results suggest that TP73-AS1 has potential as a prognostic glioma biomarker. Moreover, the knowledge that TP73-AS1 affects the glioma immune microenvironment may provide new information for the immunological research and treatment of glioma.

## INTRODUCTION

Glioma is one of the most destructive tumors with high mortality rate [1]. Therefore, it is urgent to identify prognostic markers and new therapeutic targets for glioma.

Long-noncoding RNAs (lncRNAs) are a type of transcription product with no protein coding ability. Abnormal lncRNA expression has been related to the development of glioma [2, 3]. For example, PAXIP1-AS1, as a long-noncoding RNA, was proved to facilitate progression of glioma by regulate the expression of KIF14 [4]. In addition, silencing FOXD2-AS1 could inhibit the progression of glioma and promote apoptosis of glioma cells by regulating the microRNA-98-5p/CPEB4 axis [5]. The overexpression of lncRNA LOC101928963 contributes to the proliferation of glioma cells by regulating to PMAIP1 expression [6]. The lncRNA TP73-AS1, also known as KIAA0495 and p53-dependent apoptosis modulator (PDAM), is located in human chromosomal band 1p36.32 and plays a crucial role in various carcinomas. The downregulation of TP73-AS1 has been found in oligodendromas, in which it was confirmed that the knockout of TP73-AS1 in glioma cells induced cisplatin resistance [7]. In contrast, other studies have shown that TP73-AS1 is upregulated in glioma tissues and that the knockdown of TP73-AS1 inhibits the proliferation and invasion of glioma cells [8]. Therefore, the specific role of TP73-AS1 in gliomas is not clear. In particular, immunotherapy has proven to be a very promising treatment for gliomas [9], but the relationship between TP73-AS1 and immune cell infiltration in glioma tissue has not been studied so far.

In this study, we used the CGGA RNA sequencing, TCGA low-grade and high-grade glioma dataset, and GSE16011 dataset to investigate the potential roles of TP73-AS1 in glioma. Our study suggests that the TP73-AS1 could serve as a potential prognostic biomarker and may have a nonnegligible effect on the immune microenvironment composition of glioma, which may provide new insight for the immunological research and treatment of glioma.

## RESULTS

### The expression of TP73-AS1 is correlated with poor clinicopathological features in gliomas

**Figure 1:**
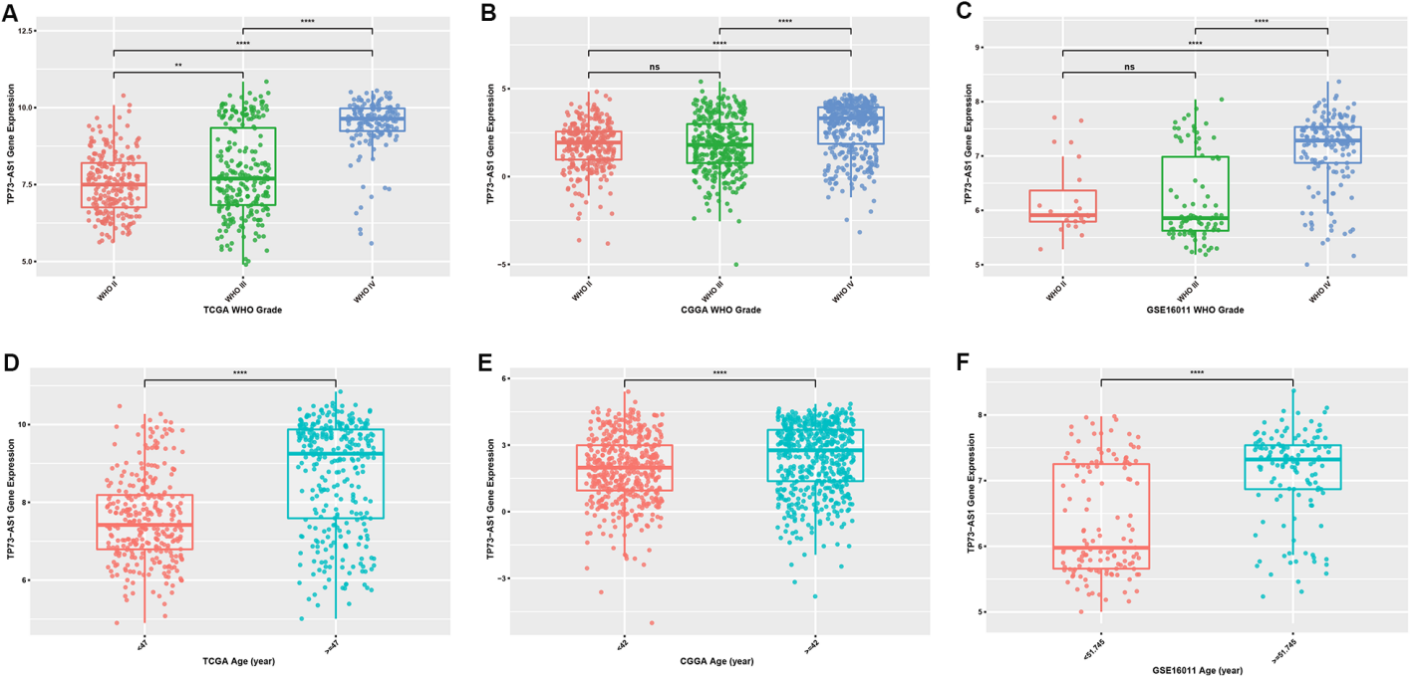
Expression levels of TP73-AS1 in gliomas with different clinicopathological features (**A**–**C**) Expression levels of TP73-AS1 in different WHO grades in the TCGA (**A**), CGGA (**B**), and GSE16011 (**C**) datasets. (**D**–**F**) Expression levels of TP73-AS1 in different age groups in the TCGA (**D**), CGGA (**E**), and GSE16011 (**F**) datasets.

To explore the difference in expression of TP73-AS1 between gliomas with different clinicopathological features, we examined the expression of TP73-AS1 in three datasets: TCGA, CGGA and GSE16011. We found that the expression of TP73-AS1 increased with the increase of WHO grades (Figure 1A–1C). Age is a risk factor for glioma patients. We found that in the three datasets, the expression of TP73-AS1 in the older age group was significantly higher than that in the younger age group (Figure 1D–1F).

**Figure 2:**
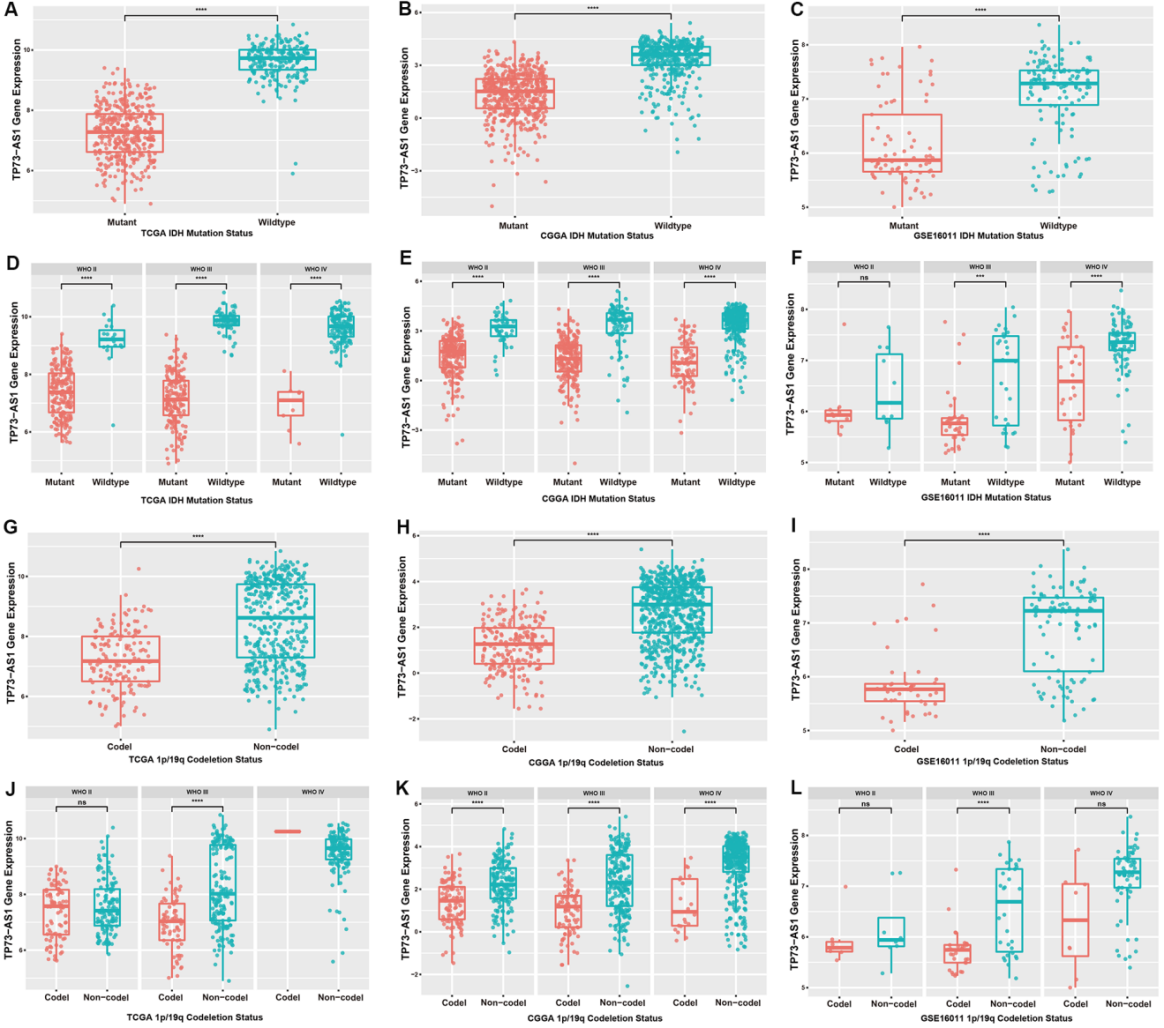
**Expression levels of TP73-AS1 in gliomas with different IDH and 1p/19q statuses.** (**A**–**C**) Expression levels of TP73-AS1 for different statuses of IDH in all WHO grades in the TCGA (**A**), CGGA (**B**), and GSE16011 (**C**) datasets. (**D**–**F**) Expression levels of TP73-AS1 for different statuses of IDH in all WHO grades in the TCGA (**D**), CGGA (**E**), and GSE16011 (**F**) datasets. (**G**–**I**) Expression levels of TP73-AS1 for different statuses of 1p/19q co-deletion in all WHO grades in the TCGA (**G**), CGGA (**H**), and GSE16011 (**I**) datasets. (**J**–**L**) Expression levels of TP73-AS1 for different statuses of 1p/19q co-deletion in all WHO grades in the TCGA (**J**), CGGA (**K**), and GSE16011 (**L**) datasets.

Considering the important influence of IDH mutation and 1p/19q chromosomal co-deletion on the prognosis of glioma patients, we evaluated the correlation between the expression of TP73-AS1 and the statuses of IDH and 1p/19q chromosome. We found the same result that TP73-AS1 expression was significantly downregulated in the IDH mutation group (Figure 2A–2F), and the 1p/19q chromosome co-deletion group in TCGA, CGGA and GSE16011 (Figure 2G–2L).

### TP73-AS1 is a risk factor for the prognosis of gliomas

**Figure 3:**
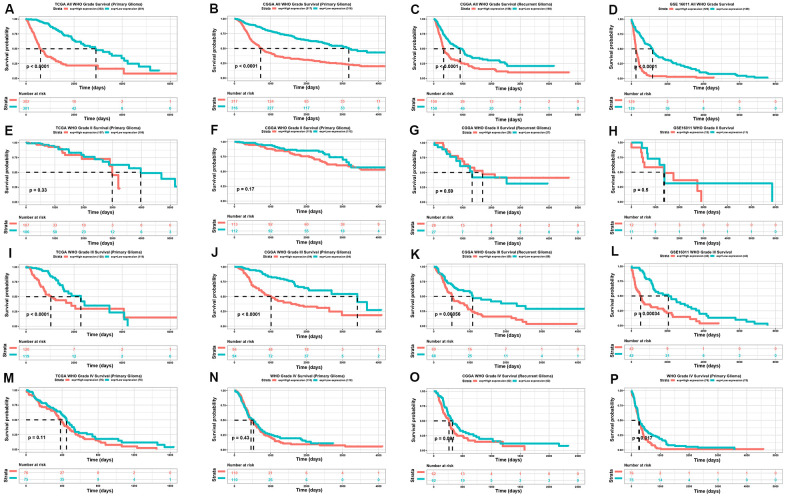
**Kaplan-meier analysis of TP73-AS1.** (**A**–**D**) Kaplan-Meier analysis of OS for the expression of TP73-AS1 in all WHO grades in the TCGA primary glioma dataset (**A**), CGGA primary glioma dataset (**B**), CGGA recurrent glioma dataset (**C**), and GSE16011 primary glioma dataset (**D**). (**E**–**H**) Kaplan-Meier analysis of OS for the expression of TP73-AS1 in WHO grade II in the TCGA primary glioma dataset (**E**), CGGA primary glioma dataset (**F**), CGGA recurrent glioma dataset (**G**), and GSE16011 primary glioma dataset (**H**). (**I**–**L**) Kaplan-Meier analysis of OS for the expression of TP73-AS1 in WHO grade III in the TCGA primary glioma dataset (**I**), CGGA primary glioma dataset (**J**), CGGA recurrent glioma dataset (**K**), and GSE16011 primary glioma dataset (**L**). (**M**–**P**) Kaplan-Meier analysis of OS for the expression of TP73-AS1 in WHO grade IV in TCGA primary glioma dataset (**M**), CGGA primary glioma dataset (**N**), CGGA recurrent glioma dataset (**O**), and GSE16011 primary glioma dataset (**P**).

The expression of TP73-AS1 is higher in glioma patients with a worse prognosis. We therefore explored the direct relationship between TP73-AS1 expression and the prognosis of glioma. We found that TP73-AS1 was a significant prognostic risk factor, including for primary gliomas in the TCGA, CGGA, and GSE16011, and recurrent gliomas in CGGA (Figure 3A–3D). We further analyzed different WHO grade of gliomas. We found that for WHO grade III gliomas, the high expression of TP73-AS1 indicated a poor prognosis for primary gliomas in the TCGA, CGGA, and GSE16011 and for recurrent gliomas of CGGA (Figure 3I–3L); however, the relation of high TP73-AS1 expression with poor prognosis was not obvious in WHO grade II (Figure 3E–3H) and WHO grade IV (Figure 3M–3P) gliomas.

### The methylation level of TP73-AS1 was negatively correlated with the expression of TP73-AS1, a favorable prognostic factor

**Figure 4:**
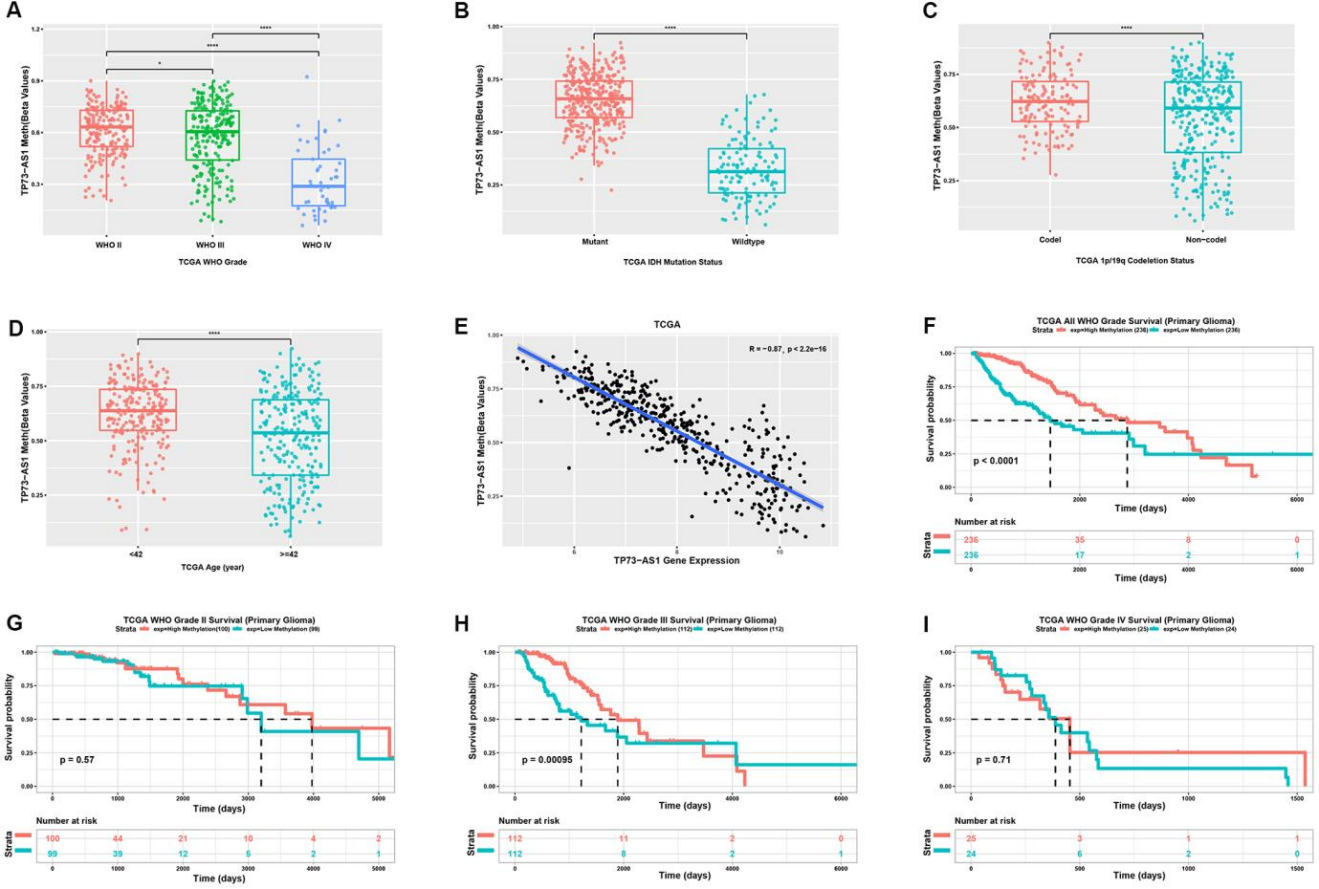
**Analysis of the TP73-AS1 methylation level.** (**A**) Methylation level of TP73-AS1 in different WHO grades in the TCGA dataset. (**B**) Methylation level of TP73-AS1 for different IDH statuses in the TCGA dataset. (**C**) Methylation level of TP73-AS1 for different 1p/19q statuses in the TCGA dataset. (**D**) Methylation level of TP73-AS1 for different age groups in the TCGA dataset. (**E**) Pearson’s correlation between the methylation level and mRNA expression of TP73-AS1. (**F**) Kaplan-Meier analysis of OS for the methylation level of TP73-AS1 for all WHO grades in the TCGA primary glioma dataset. (**G**) Kaplan-Meier analysis of OS for the methylation level of TP73-AS1 in WHO grade II in the TCGA primary glioma dataset. (**H**) Kaplan-Meier analysis of OS for the methylation level of TP73-AS1 in WHO grade III in the TCGA primary glioma dataset. (**I**) Kaplan-Meier analysis of OS for the methylation level of TP73-AS1 in WHO grade IV in the TCGA primary glioma dataset.

In general, promoter methylation regulates gene expression. We analyzed the methylation levels of TP73-AS1 in gliomas with different clinicopathological features. We found that the methylation level of TP73-AS1 decreased with an increase in WHO grade in the TCGA database (Figure 4A). IDH mutation (Figure 4B) and 1p/19q co-deletion (Figure 4C) led to high levels of TP73-AS1 methylation. Moreover, the methylation level of TP73-AS1 was decreased in the older age group (Figure 4D). We confirmed that the methylation level of the TP73-AS1 promoter was negatively correlated with the expression level of TP73-AS1, suggesting that the methylation of the TP73-AS1 promoter led to a decrease in TP73-AS1 expression (Figure 4E). Survival analysis showed that the methylation level of TP73-AS1 was a favorable prognostic factor, especially in WHO grade III gliomas (Figure 4F–4I).

### Functional enrichment analysis of TP73-AS1

**Figure 5:**
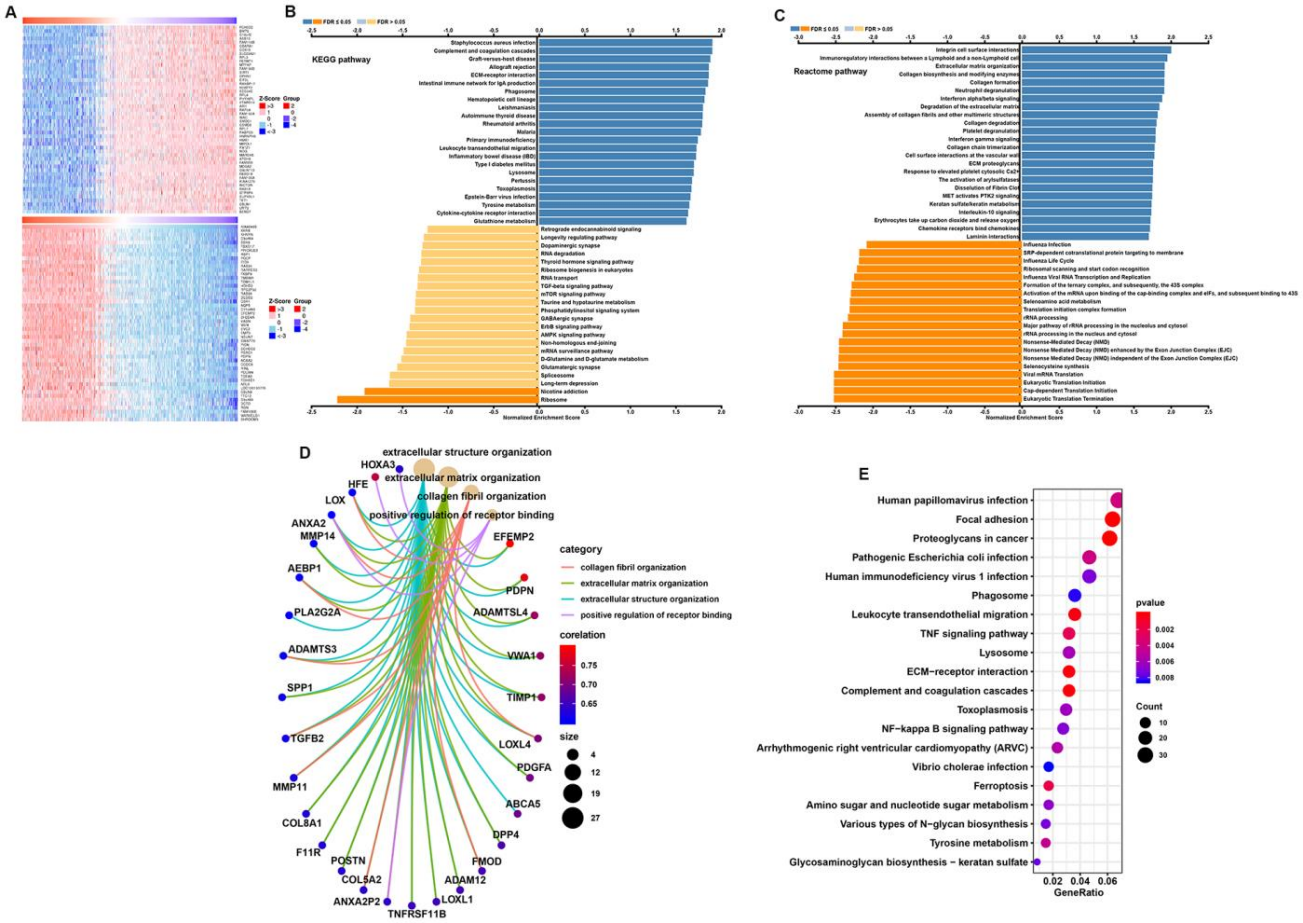
**Function and pathway enrichment analysis for TP73-AS1.** (**A**) Heatmaps normalized using the Z-scores of the top 50 genes positively and negatively correlated with TP73-AS1. (**B**, **C**) Significant GSEA results of TP73-AS1, including KEGG pathways (**B**) and Reactome pathways (**C**). (**D**) Significant Gene Ontology terms of TP73-AS1. (**E**) Significant KEGG pathways of TP73-AS1.

To further study the function of TP73-AS1, we used the TCGA based database Linkedomics to perform a correlation analysis of TP73-AS1. Two heatmaps were constructed to illustrate the genes whose expression was most positively and negatively correlated with that of TP73-AS1 (Figure 5A). GSEA showed that TP73-AS1 probably participates in KEGG pathways, including those involving *Staphylococcus aureus* infection, complement and coagulation cascades, and Reactome pathways, including integrin cell surface interactions and immunoregulatory interactions between a Lymphoid and a non-Lymphoid cell. The genes whose expression was negatively related with that of TP73-AS1 were enriched in KEGG pathways, including those associated with the ribosome and nicotine addiction and Reactome pathways, including eukaryotic translation termination and cap-dependent translation initiation pathways (Figure 5B–5C).

To further explore potential functional pathways associated with TP73-AS1, we conducted a functional pathway analysis based on the most positively correlated genes identified in the abovementioned analysis (Pearson r>0.5, P<0.01) using the “org.Hs.eg.db,” “clusterProfiler,” and “enrichplot” R packages. These genes were mainly associated with extracellular structure organization, extracellular matrix organization, collagen fibril organization, and positive regulation of receptor binding in GO (Gene Ontology) categories (Figure 5D). KEGG pathway analysis indicated that the pathways in which the most highly-correlated genes were enriched involved focal adhesion, proteoglycans in cancer, ECM−receptor interactions, and leukocyte transendothelial migration (Figure 5F).

### Tumor immune microenvironment analysis

**Figure 6:**
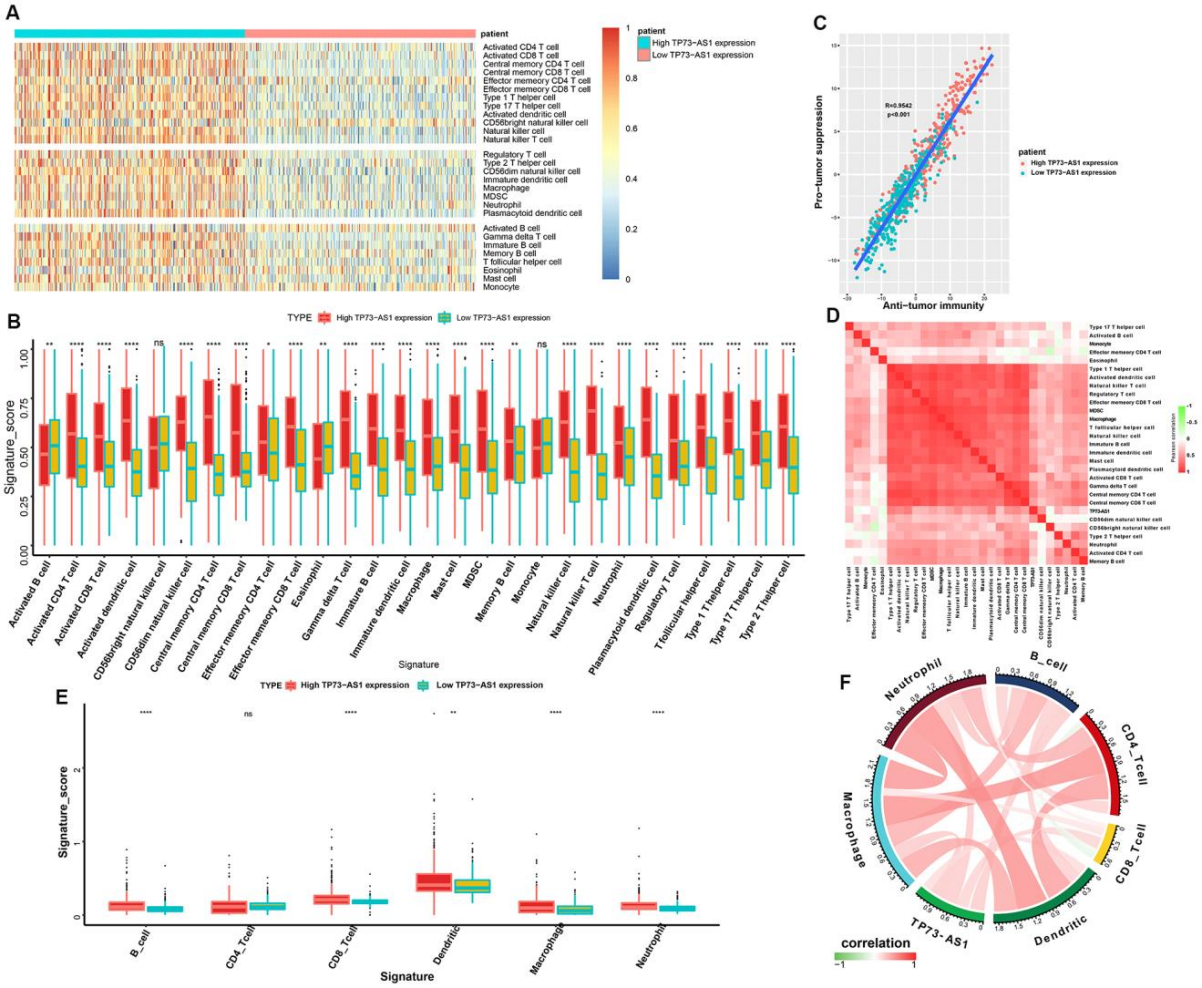
**Tumor immune microenvironment analysis for TP73-AS1.** (**A**) Heatmap normalized using the Z-scores of the 28 tumor-infiltrating immune cell populations. (**B**) The signature scores (ssGSEA scores) of 28 infiltrating immune cells in the high and low TP73-AS1 expression groups. (**C**) Pearson’s correlation between the pro-tumor suppression score and anti-tumor immunity score. (**D**) The correlation heatmaps of the TP73-AS1 expression levels and infiltration scores (ssGSEA scores) of different immune cells. (**E**) The signature scores (Timer) of six infiltrating immune cells in high and low TP73-AS1 expression groups. (**F**) The correlation circle diagram of the TP73-AS1 expression and infiltration scores (Timer) of different immune cells.

The tumor microenvironment (TME), especially the tumor immune microenvironment, is important for the survival of tumor patients. To evaluate the populations of various immune cells, we employed single-sample gene set enrichment analysis (ssGSEA; https://www.genepattern.org/modules/docs/ssGSEAProjection/4) to determine the relative infiltration level of 28 immune cells (Figure 6A). We made two major observations: (1) The high TP73-AS1 group had more complex immune cell components than the low TP73-AS1 group, including cells exhibiting anti-tumor reactivity, such as activated CD4 T cells, activated dendritic cells, central memory CD4 T cells, central memory CD8 T cells, natural killer cells, and natural killer T cells, and cells involved in tumor suppression, such as immature dendritic cells, MDSCs, plasmacytoid dendritic cells, regulatory T cells, and type 2 T helper cells (Figure 6B). (2) The correlation analysis revealed that the infiltration level of these two categories of immune cells were positively correlated (Figure 6C). This result revealed a feedback mechanism that anti-tumor inflammation could promote the recruitment or differentiation of immunosuppressive cells. Correlation analysis showed that TP73-AS1 expression was positively correlated with the infiltration scores of most immune cells (Figure 6D). To further verify the results, we used the Timer database (https://cistrome.shinyapps.io/timer/) to analyze the infiltration scores of immune cells in glioma tissue. We came to the same conclusion, that the high TP73-AS1 group had more complex immune cell components (Figure 6E) and that TP73-AS1 was positively correlated with the infiltration scores of most immune cells (Figure 6F).

### Data analysis from the hospital

**Figure 7:**
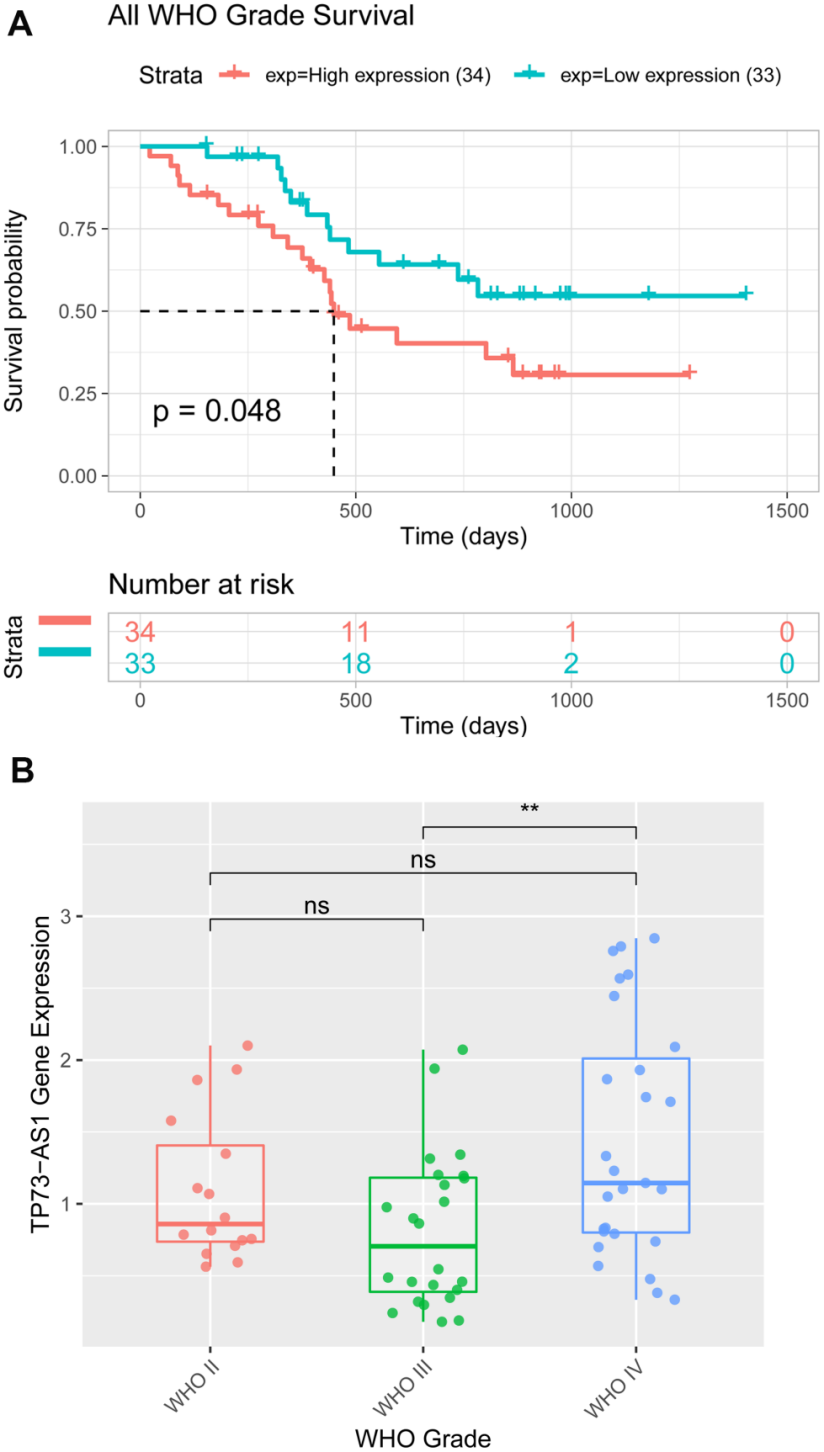
**Expression and Kaplan-Meier analyses of TP73-AS1 in patients with glioma from the hospital.** (**A**) The expression value of TP73-AS1 in glioma samples of patients from the hospital with different WHO grades. (**B**) Kaplan-Meier analysis of OS for the expression of TP73-AS1 in glioma samples of patients from the hospital with all WHO grades.

We found that TP73-AS1 was differentially expressed in gliomas with different clinicopathological features in the TCGA, CGGA, and GEO glioma datasets. The overexpression of TP73-AS1 was associated with poor clinical features, including age, stage, IDH mutation status, 1p/19q co-deletion status, and overall survival. RT-PCR was performed to detect the expression of the TP73-AS1 protein in 67 glioma tissues of different grades from our hospital. The results showed higher expression of TP73-AS1 in glioma samples of WHO grade IV than in glioma samples of WHO grade IV (p=0.002) (Figure 7A), and high TP73-AS1 expression predicted a poorer prognosis than low TP73-AS1 expression (P=0.048) (Figure 7B); these findings agreed with our preliminary results. We also found that TP73-AS1 expression levels were significantly correlated with the WHO grade of gliomas (p <0.0001), IDH1 mutation (p=0.0035), and 1p/19q co-deletion state (p=0.0119) (Table 1).

**Table 1:** Correlation between the expression of TP73-AS1 and clinicopathological characteristics in glioma tissue.

**Characteristic**		**Expression level of TP73-AS1**		
	**n**	**Low (n)**	**High (n)**	**P-value**
Age (years)				
>50	44	18	26	0.0588
≤50	23	15	8	
Gender				
Male	35	18	17	0.7096
Female	32	15	17	
Tumor location				
Frontal lobe	30	15	15	0.4931
Temporal lobe	21	12	9	
Others	16	6	10	
Tumor diameter				
<3cm	30	16	14	0.411
>3cm	37	16	21	
KPS				
<80	32	14	18	0.3889
>80	35	19	16	
Extent of resection				
Gross total resection	34	19	15	0.2706
Subtotal resection	33	14	19	
IDH1				
Mutation	23	17	6	**0.0035**
Wild	44	16	28	
MGMT methylation				
Positive	41	23	18	0.1594
Negative	26	10	16	
1p/19q codeletion				
Exist	19	14	5	**0.0119**
Without	48	19	29	
WHO grade				
II	16	14	2	**<0.0001**
III	24	14	10	
IV	27	5	22	

## DISCUSSION

Malignant progression and high recurrence rate make glioma the most lethal primary brain tumor [10, 11]. Disorders related to lncRNAs may contribute to the pathogenesis of glioma [12–14]. In this study, we aimed to assess whether TP73-AS1 could function in the development of glioma.

It was reported that TP73-AS1 is associated to aggressiveness in glioblastoma and promotes tumorigenicity of medulloblastoma cells [15, 16]. To ascertain the role of TP73-AS1 in glioma more clearly, we collected TCGA, CGGA, and GSE16011 glioma data to explore the clinicopathological features related toTP73-AS1. We found that level of TP73-AS1 increased with the glioma WHO grade and age of patients in all three datasets. IDH mutation and 1p/19q chromosome co-deletion have a huge influence on the prognosis of glioma patients [17–20]. TP73-AS1 expression was significantly downregulated in the IDH mutation group and 1p/19q chromosome co-deletion groups in the TCGA, CGGA, and GSE16011, indicating that TP73-AS1 is a poor prognostic factor for glioma, which was consistent with previous reports [15, 16]. Further analysis showed that TP73-AS1 is a poor prognostic factor for glioma, especially WHO grade III glioma in the TCGA, CGGA, and GSE16011 datasets. In contrast, the methylation level of TP73-AS1 was found to be a valuable prognostic factor for glioma, especially for WHO grade III glioma.

To further study the function of TP73-AS1, we divided the TCGA glioma samples into a high TP73-AS1 group and a low TP73-AS1 group, using the median expression of TP73-AS1 in the samples. The GSEA results showed that KEGG pathways, including those related to Staphylococcus aureus infection, complement and coagulation cascades, and Reactome pathways, including integrin cell surface interaction pathways and pathways related to immunoregulatory interactions between lymphoid and nonlymphoid cells, were significantly enriched in the high TP73-AS1 group, while KEGG pathways, including those related to the ribosome and nicotine addiction, and Reactome pathways, including eukaryotic translation termination and cap-dependent translation initiation pathways, were significantly enriched in the low TP73-AS1 group.

Immune cells in the tumor microenvironment are a vital element of tumor tissue [21–23]. Our results demonstrated that the high TP73-AS1 group was significantly associated with many immune-related signatures. By investigating the regulation of immune cell populations, we made two major observations: (1) The high-risk group had more complex immune cell components than the low-risk group, including cells exhibiting anti-tumor reactivity, such as activated CD4 T cells, activated dendritic cells, central memory CD4 T cells, central memory CD8 T cells, natural killer cells, and natural killer T cells, and cells with tumor suppression activity, such as immature dendritic cells, MDSCs, plasmacytoid dendritic cells, regulatory T cells, and type 2 T helper cells. (2) The correlation analysis revealed that the infiltration level of these two categories of immune cells were positively correlated. Correlation analysis based on two different analytical methods showed that TP73-AS1 was positively correlated with the infiltration scores of most of the immune cells. These results suggest that TP73-AS1 has a non-negligible effect on the immune microenvironment of glioma.

Overall, our research indicated that TP73-AS1 is a reliable marker for predicting the overall survival of glioma patients and that TP73-AS1 has a non-negligible effect on the immune microenvironment of glioma.

## MATERIALS AND METHODS

### Data collection

The expression data and corresponding clinical information of glioma patients were obtained from (1) the TCGA dataset (https://xenabrowser.net/), which included expression data and data on the clinicopathological features of 664 patients; (2) CGGA RNA-seq data (http://cgga.org.cn/), which included expression data and data on the clinicopathological features of 984 patients; and (3) GSE16011 (https://www.ncbi.nlm.nih.gov), which included expression data and data on the clinicopathological features of 258 patients. This study was approved by the ethics committee of the Zhejiang Cancer Hospital. Written informed consent was obtained from all participants in this study.

### qRT-PCR

TRIzol reagent (Life Technologies) and RevertAid First Strand cDNA Synthesis Kit (Thermo Scientific) were used to extract Total RNA and synthesize cDNA according to the manufacturer’s protocol. qRT-PCR analysis was performed using the standard protocol of the SYBR Select Master Mix kit. GAPDH was included as an internal reference gene. The primers used were as follows:

TP73-AS1-F 5ʹ - CCGTGGTTAGCGTGGACATCAG – 3ʹ

TP73-AS1-R 5ʹ - TGGGTGACAGGGCAAGACTCC – 3ʹ

GAPDH-F 5ʹ - CATGAGAAGTATGACAACAGCCT – 3ʹ

GAPDH-R 5ʹ - AGTCCTTCCACGATACCAAAGT – 3ʹ

### Differential expression analysis and survival analysis

To investigate the expression of TP73-AS1, we used the R packages ggplot2, ggpubr, and grid Extra in the R software (3.6.2) to study gene expression patterns in gliomas with different clinicopathological features. For survival analysis, the R packages survminer, survival, ggpubr, and gridExtra were employed. The age segmentation value of each dataset was the median age of patients in the data set.

### Gene set enrichment analyses and enrichment analysis

Data from the TCGA dataset were used for gene set enrichment analyses and enrichment analysis. GO analysis was performed using the EnrichGO function in the clusterProfiler R package, with the following parameters: ont = “all” pvalue-Cutoff = 0.05 and qvalue-Cutoff = 0.05. KEGG analysis was performed using the EnrichKEGG function of the R package “clusterProfiler,” with the following parameters: keyType = “kegg” pvalue-Cutoff = 0.05 and qvalue-Cutoff = 0.05. Gene set enrichment analyses were performed using the Linkedomics online dataset (http://www.linkedomics.org/login.php).

### Immune cell infiltration

Single-sample GSEA was used to evaluate the infiltration level of 28 immune cell types. Immune cell signatures were obtained from a published paper [24]. The infiltration levels of immune cell types were represented by an enrichment score in the ssGSEA analysis of the GSVA R package. The ssGSEA score was normalized to unity distribution, with zero as the minimum and one as the maximum score for each immune cell type.

### Ethics approval and consent to participate

Additional informed consent was obtained from all participants whose identifying information was included in this article.

### Data availability statement

The datasets generated and/or analyzed during the current study are available from the corresponding author upon reasonable request.

## References

[1] Tiwari V, Daoud EV, Hatanpaa KJ, Gao A, Zhang S, An Z, Ganji SK, Raisanen JM, Lewis CM, Askari P, Baxter J, Levy M, Dimitrov I, et al. Glycine by MR spectroscopy is an imaging biomarker of glioma aggressiveness. Neuro Oncol. 2020; 22:1018–29. 10.1093/neuonc/noaa034 32055850 PMC7339885

[2] Yang J, Sun G, Hu Y, Yang J, Shi Y, Liu H, Li C, Wang Y, Lv Z, Niu J, Liu H, Shi X, Wang H, et al. Extracellular vesicle lncRNA metastasis-associated lung adenocarcinoma transcript 1 released from glioma stem cells modulates the inflammatory response of microglia after lipopolysaccharide stimulation through regulating miR-129-5p/high mobility group Box-1 protein axis. Front Immunol. 2020; 10:3161. 10.3389/fimmu.2019.03161 32117213 PMC7020807

[3] Ding J, Zhang L, Chen S, Cao H, Xu C, Wang X. lncRNA CCAT2 enhanced resistance of glioma cells against chemodrugs by disturbing the normal function of miR-424. Onco Targets Ther. 2020; 13:1431–45. 10.2147/OTT.S227831 32110042 PMC7034969

[4] Xu H, Zhao G, Zhang Y, Jiang H, Wang W, Zhao D, Yu H, Qi L. Long non-coding RNA PAXIP1-AS1 facilitates cell invasion and angiogenesis of glioma by recruiting transcription factor ETS1 to upregulate KIF14 expression. J Exp Clin Cancer Res. 2019; 38:486. 10.1186/s13046-019-1474-7 31823805 PMC6902534

[5] Gu N, Wang X, Di Z, Xiong J, Ma Y, Yan Y, Qian Y, Zhang Q, Yu J. Silencing lncRNA FOXD2-AS1 inhibits proliferation, migration, invasion and drug resistance of drug-resistant glioma cells and promotes their apoptosis via microRNA-98-5p/CPEB4 axis. Aging (Albany NY). 2019; 11:10266–83. 10.18632/aging.102455 31770107 PMC6914387

[6] Zheng YJ, Liang TS, Wang J, Zhao JY, Yang DK, Liu ZS. Silencing lncRNA LOC101928963 inhibits proliferation and promotes apoptosis in spinal cord glioma cells by binding to PMAIP1. Mol Ther Nucleic Acids. 2019; 18:485–95. 10.1016/j.omtn.2019.07.026 31670198 PMC6838552

[7] Pang JC, Li KK, Lau KM, Ng YL, Wong J, Chung NY, Li HM, Chui YL, Lui VW, Chen ZP, Chan DT, Poon WS, Wang Y, et al. KIAA0495/PDAM is frequently downregulated in oligodendroglial tumors and its knockdown by siRNA induces cisplatin resistance in glioma cells. Brain Pathol. 2010; 20:1021–32. 10.1111/j.1750-3639.2010.00405.x 20477830 PMC8094719

[8] Zhang R, Jin H, Lou F. The long non-coding RNA TP73-AS1 interacted with miR-142 to modulate brain glioma growth through HMGB1/RAGE pathway. J Cell Biochem. 2018; 119:3007–16. 10.1002/jcb.26021 28379612

[9] Mosaheb MM, Dobrikova EY, Brown MC, Yang Y, Cable J, Okada H, Nair SK, Bigner DD, Ashley DM, Gromeier M. Genetically stable poliovirus vectors activate dendritic cells and prime antitumor CD8 T cell immunity. Nat Commun. 2020; 11:524. 10.1038/s41467-019-13939-z 31988324 PMC6985231

[10] Nejo T, Yamamichi A, Almeida ND, Goretsky YE, Okada H. Tumor antigens in glioma. Semin Immunol. 2020; 47:101385. 10.1016/j.smim.2020.101385 32037183

[11] Gutmann DH. The sociobiology of brain tumors. Adv Exp Med Biol. 2020; 1225:115–25. 10.1007/978-3-030-35727-6_8 32030651

[12] Zhang Y, Mou C, Shang M, Jiang M, Xu C. Long noncoding RNA RP11-626G11.3 promotes the progression of glioma through miR-375-SP1 axis. Mol Carcinog. 2020; 59:492–502. 10.1002/mc.23173 32128886

[13] Wang S, Qi Y, Gao X, Qiu W, Liu Q, Guo X, Qian M, Chen Z, Zhang Z, Wang H, Xu J, Xue H, Guo X, et al. Hypoxia-induced lncRNA PDIA3P1 promotes mesenchymal transition via sponging of miR-124-3p in glioma. Cell Death Dis. 2020; 11:168. 10.1038/s41419-020-2345-z 32127518 PMC7054337

[14] Huang W, Shi Y, Han B, Wang Q, Zhang B, Qi C, Liu F. LncRNA GAS5-AS1 inhibits glioma proliferation, migration, and invasion via miR-106b-5p/TUSC2 axis. Hum Cell. 2020; 33:416–26. 10.1007/s13577-020-00331-z 32072565

[15] Varon M, Levy T, Mazor G, Ben David H, Marciano R, Krelin Y, Prasad M, Elkabets M, Pauck D, Ahmadov U, Picard D, Qin N, Borkhardt A, et al. The long noncoding RNA TP73-AS1 promotes tumorigenicity of medulloblastoma cells. Int J Cancer. 2019; 145:3402–13. 10.1002/ijc.32400 31081944

[16] Mazor G, Levin L, Picard D, Ahmadov U, Carén H, Borkhardt A, Reifenberger G, Leprivier G, Remke M, Rotblat B. The lncRNA TP73-AS1 is linked to aggressiveness in glioblastoma and promotes temozolomide resistance in glioblastoma cancer stem cells. Cell Death Dis. 2019; 10:246. 10.1038/s41419-019-1477-5 30867410 PMC6416247

[17] Barritault M, Picart T, Poncet D, Fenouil T, d’Hombres A, Gabut M, Guyotat J, Jouanneau E, Ameli R, Joubert B, Streichenberger N, Vasiljevic A, Honnorat J, et al. Avoiding new biopsies by identification of IDH1 and TERT promoter mutation in nondiagnostic biopsies from glioma patients. Neurosurgery. 2020; 87:E513–19. 10.1093/neuros/nyaa025 32107549

[18] Wu H, Tong H, Du X, Guo H, Ma Q, Zhang Y, Zhou X, Liu H, Wang S, Fang J, Zhang W. Vascular habitat analysis based on dynamic susceptibility contrast perfusion MRI predicts IDH mutation status and prognosis in high-grade gliomas. Eur Radiol. 2020; 30:3254–65. 10.1007/s00330-020-06702-2 32078014

[19] Chen SC, Lo CM, Wang SH, Su EC. RNA editing-based classification of diffuse gliomas: predicting isocitrate dehydrogenase mutation and chromosome 1p/19q codeletion. BMC Bioinformatics. 2019 (Suppl 19); 20:659. 10.1186/s12859-019-3236-0 31870275 PMC6929429

[20] Murakami C, Ikota H, Nobusawa S, Nakata S, Yamazaki T, Hashiba Y, Hirato J, Yokoo H. Oligodendroglioma showing pleomorphic xanthoastrocytoma-like perivascular microlesion: with IDH1, TERT promoter mutation and 1p/19q codeletion detected in both components. Pathol Int. 2020; 70:40–46. 10.1111/pin.12880 31855307

[21] Greten FR, Grivennikov SI. Inflammation and cancer: triggers, mechanisms, and consequences. Immunity. 2019; 51:27–41. 10.1016/j.immuni.2019.06.025 31315034 PMC6831096

[22] Mukaida N, Sasaki SI, Baba T. CCL4 signaling in the tumor microenvironment. Adv Exp Med Biol. 2020; 1231:23–32. 10.1007/978-3-030-36667-4_3 32060843

[23] Zewdu A, Casadei L, Pollock RE, Braggio D. Adipose tumor microenvironment. Adv Exp Med Biol. 2020; 1226:73–86. 10.1007/978-3-030-36214-0_6 32030677

[24] Charoentong P, Finotello F, Angelova M, Mayer C, Efremova M, Rieder D, Hackl H, Trajanoski Z. Pan-cancer immunogenomic analyses reveal genotype-immunophenotype relationships and predictors of response to checkpoint blockade. Cell Rep. 2017; 18:248–62. 10.1016/j.celrep.2016.12.019 28052254

